# Household Food Insecurity and Feeding Practices in Brazilian Children < 5 Years: Results From Brazilian National Survey on Child Nutrition

**DOI:** 10.1111/jhn.70146

**Published:** 2025-10-28

**Authors:** Juliana Vieira de Castro Mello, Raquel Machado Schincaglia, Natália Oliveira, Nadya Helena Alves‐Santos, Paula Normando, Dayana Rodrigues Farias, Elisa Maria de Aquino Lacerda, Inês Rugani Ribeiro de Castro, Gilberto Kac

**Affiliations:** ^1^ Nutritional Epidemiology Observatory, Institute of Nutrition Josué de Castro, Federal University of Rio de Janeiro, University City ‐ Ilha do Fundão Rio de Janeiro Brazil; ^2^ Faculty of Nutrition, Federal University of Goiás, Setor Leste Universitário Goiânia Goiás Brazil; ^3^ Institute of Health Sciences, Federal University of Pará, Campus Universitário do Guamá, Setor Saúde Belém Brazil; ^4^ Institute of Nutrition Josué de Castro, Federal University of Rio de Janeiro, University City ‐ Ilha do Fundão Rio de Janeiro Brazil; ^5^ Institute of Nutrition, State University of Rio de Janeiro Rio de Janeiro Brazil

**Keywords:** complementary feeding, feeding behaviour, food security, infant, preschool child

## Abstract

**Background:**

In 2023, food insecurity (FI) impacted households with children under 5 years old (< 5 y), where FI reached 37.5%. Establishing the link between feeding practices and FI in household surveys is essential to understanding children's dietary habits. However, this association has not yet been examined in Brazil using nationally representative data. This study aims to describe the prevalence of FI by sociodemographic variables and to estimate its association with feeding practices in Brazilian children < 5 y.

**Methodology:**

This study analysed data from the Brazilian National Survey on Child Nutrition (ENANI‐2019), a household‐based population evaluating 14,558 children < 5 y. FI was assessed using the Brazilian Food Insecurity Scale. Feeding practices were defined using national and international indicators/recommendations for each age group, using a structured questionnaire about food intake the day before the interview (< 6 months: early introduction of complementary feeding; 6–23 months: minimum dietary diversity (MDD), egg and/or flesh foods, and zero consumption of vegetables or fruits; 24–59 months: beans, vegetables, fruit, sweetened beverage and ultra‐processed food consumption). Logistic regression was performed to estimate the association between FI and feeding practices, with p‐values adjusted for multiple testing using the Benjamini‐Hochberg method.

**Results:**

FI was not associated with the early introduction of complementary feeding. Children aged 6–23 months experiencing severe FI had a 60% lower chance of meeting MDD (OR = 0.40 [95% CI: 0.21; 0.77]) and were 2.48 times more likely to consume zero vegetables or fruit (OR = 2.48 [95% CI: 1.31;4.69]) compared to those in food‐secure households. Children aged 24–59 months living in severe FI had a 53% lower chance of consuming vegetables than their food‐secure counterparts (OR = 0.47 [95% CI: 0.30;0.73]).

**Conclusions:**

We found socioeconomic disparities across FI levels, with poorer feeding practices in food‐insecure households, except among children under 6 months. Severe FI was associated to reduced fruit and vegetable intake and lower dietary diversity. Addressing FI requires policies that ensure food quantity and quality, prioritising low‐income and underserved families. Future research should explore structural determinants and assess targeted interventions to improve child nutrition in the context of FI.

## Summary

- We observed differences in the socioeconomic indicators (region of residence, number of residents per bedroom, and per capita income) across FI categories.
- Food‐insecure households had significant disparities in feeding practices, except for those aged < 6 months.
- Children aged 6–23 months experiencing severe FI had a 60% lower chance of meeting minimum dietary diversity (OR = 0.40 [95% CI: 0.21; 0.77]) and were 2.48 times more likely to consume zero vegetables or fruit (OR = 2.48 [95% CI: 1.31;4.69]) compared to those in food‐secure households.
- Children aged 24–59 months living in severe FI had a 53% lower chance of consuming vegetables than their food‐secure counterparts (OR = 0.47 [95% CI: 0.30;0.73]).

## 1 Introduction

According to the Food and Agriculture Organisation of the United Nations (FAO), food security exists when all people, at all times, have physical and economic access to sufficient, safe, and nutritious food that meets their dietary needs and food preferences for an active and healthy life [1]. Food insecurity (FI) refers to the inability of individuals or households to access adequate food, and it is usually classified as mild, moderate, or severe. Mild FI involves concerns about food access and reduced food quality. Moderate FI includes reduced food intake among adults and disrupted eating patterns. Severe FI affects children, involving skipped meals and episodes of hunger, such as going a whole day without eating due to a lack of resources [2, 3, 4].

Around 30% of the world′s population was in moderate and severe FI in 2022. In Latin America and the Caribbean, FI prevalence has increased from 27.3% in 2015 to 31.5% in 2019, reaching 37.5% of the population in 2022 [5]. In Brazil, FI reached 59% in 2022, and the prevalence of its severe form, which can be considered hunger, increased from 9% to 16%, representing 33 million Brazilians [6]. Evidence from Brazilian studies highlights that low income, limited education, poor housing conditions, and restricted access to basic sanitation are key determinants of FI. A systematic review analysing 18 studies conducted between 2003 and 2018 confirmed that lower per capita income and lower educational attainment of the household head are consistently associated with higher levels of FI, reinforcing the role of structural socioeconomic inequalities in shaping food access and dietary practices [7].

In 2023, the prevalence of any level of FI decreased to 27.6%, with a higher prevalence in Brazilian households with children under 5 years of age (< 5 y), where 37.5% were experiencing some degree of FI, and 4.5% of these households were in severe FI [8]. Living in contexts of FI can seriously compromise health during childhood, a critical stage when food and nutrition are typically prioritised within households [9, 10, 11, 12]. Interventions during the early years of life are essential for young children to achieve their developmental potential. FI can lead to nutritional deficiencies, and undernutrition remains a serious challenge in developing countries, compromising young children's survival, growth, and development [13]. Neglect of these basic needs reflects a condition of severe deprivation [3, 14]. Evidence indicates that children living in food‐insecure households have a higher prevalence of hospitalisations due to pneumonia or diarrhoea [12], as well as impaired development across multiple domains, with marked effects on cognitive, motor, behavioural, and academic outcomes [9, 10].

Indicators of feeding practices are commonly used to characterise children′s diets. It is globally recognised that exclusive breastfeeding until 6 months is recommended, as it reduces morbidity from gastrointestinal infections [15] and lowers infant mortality rates [16]. Therefore, a key recommendation for this age group is to emphasise the importance of avoiding the early introduction of complementary feeding. In 2021, the World Health Organisation (WHO) and the United Nations International Children's Emergency Fund introduced a set of new and updated indicators to assess infant and young child feeding practices at the household level for children aged 6–23 months. These include minimum dietary diversity (MDD), egg and/or flesh food consumption, sweet beverage consumption, unhealthy food consumption, and zero vegetable or fruit consumption [17]. For children between 2 and 10 years, there is a lack of concrete global recommendations for assessing feeding practices. However, according to the Brazilian Dietary Guidelines, a healthy diet for this age group includes consuming beans, fruits, and vegetables daily and avoiding ultra‐processed foods and sweetened beverages [18, 19].

Some studies found an association between FI and feeding practices in children 6–23 months in low‐ and middle‐income countries such as Ghana, Pakistan, Kenya, and Mexico. Children in households living in food security had a higher prevalence of meeting MDD than those in FI [20, 21, 22]. It was also observed that children aged 6–11 years from food‐insecure households have greater intakes of total energy and calories from added sugar [23]. Establishing the association between feeding practices and FI in household‐based population surveys is essential for understanding children's dietary habits in food‐insecure households. This understanding can inform targeted actions beyond general healthy eating recommendations, focusing on promoting accessible and affordable dietary alternatives. However, to date, this analysis has not yet been conducted in Brazil using national data. Therefore, this study aims to describe the prevalence of FI by sociodemographic variables and to analyse the association between FI levels and feeding practices in Brazilian children < 5 y.

## 2 Material and Methods

### 2.1 Study Design, Population, and Data Collection

Brazilian National Survey on Child Nutrition (ENANI‐2019) is a household‐based population survey that evaluated 14,558 children < 5 y [24]. The sample was selected using a probabilistic, stratified, and clustered design. It was stratified by Brazil′s five major geographic regions and further grouped by census tracts. A total of 1500 tracts were selected with probability proportional to the number of children under 5 years of age. In each tract, up to 10 households were included, totalling 15,000 households. Sampling weights were calculated to account for the complex design and ensure national and regional representativeness. The design allowed us to generate estimates representing Brazil′s five geographic regions, child's sex, and age groups. More details of the sample design have been published previously [25].

Data collection occurred from February 2019 to March 2020 and was conducted by 323 trained interviewers. A questionnaire was administered covering various aspects related to households, mothers/caregivers, and children. It included detailed questions on socioeconomic conditions, household composition, maternal/caregiver education, employment status, access to basic services, and child health and nutrition. The general methodological aspects of ENANI‐2019 have been published previously [24].

### 2.2 Food Insecurity

A structured questionnaire, slightly adapted from the Brazilian Food Insecurity Scale (EBIA), was used to estimate the FI. The adaptation involved specific wording changes only and was carefully analysed to ensure data quality and consistency with the original scale′s validation [3, 26].

This instrument comprises 14 items with responses of ‘yes’, ‘no’ or ‘does not know/chose not to respond’. The responses to the latter option represented < 1% and were imputed. This scale allows the estimation of household FI across three levels, predicated on the number of affirmative responses: 1–5 categorised as mild FI, 6−9 as moderate FI, and > 10 as severe FI. If the answer was ‘no’ to all questions, the household was classified as being in food security [3].

### 2.3 Feeding Practices

Feeding practices were defined using national and international indicators, calculated using a questionnaire based on the Brazilian Food and Nutrition Surveillance System instrument [27]. This structured questionnaire included 40 questions on food intake on the day before the interview, with response options of ‘yes’, ‘no’ or ‘does not know/refuse to answer’. The responses to the latter option represented < 1% and were imputed. Age‐specific indicators (< 6 months, 6–23 months, and 24–59 months) were used.

For children < 6 months, the introduction of any food other than breast milk and/or infant formula was considered an early introduction of complementary feeding, using 23 questions. For children aged 6−23 months, the indicators proposed by the WHO were used [17]. We could estimate three of the nine WHO complementary feeding indicators based on the data collected in our questionnaire. These included MDD, which was considered when the child consumed at least five out of the eight food groups: (1) breast milk; (2) grains, roots, tubers, and plantains; (3) pulses (beans, peas, lentils), nuts, and seeds; (4) dairy products (milk, infant formula, yogurt, cheese); (5) flesh foods (meat, fish, poultry, organ meats); (6) eggs; (7) vitamin‐A rich fruits and vegetables; and (8) other fruits and vegetables. Additionally, the indicator for consumption of eggs and/or flesh foods was based on consumption of the previously described food groups 5 and 6. Finally, zero consumption of vegetables or fruits was determined based on food groups 7 and 8 [17].

For children aged 24−59 months, in the absence of clear global recommendations for assessing these feeding practices, we used food markers of consumption according to the Brazilian Dietary Guidelines [18]. Since these guidelines recommend the daily consumption of beans or other legumes, vegetables, and fruits while advising the intake against consuming sweetened beverages and ultra‐processed foods (not including sweetened beverages), we used this as food markers, using 17 questions. The NOVA classification was applied to categorise ultra‐processed food, excluding sugary beverages, which were also considered separated in the marker assessed [28].

### 2.4 Other Variables

We collected information about the Brazilian region where that household was located (North, Northeast, Southeast, South, and Midwest). Access to basic sanitation was measured considering the simultaneous presence of water supply, sewage, and solid waste management. Maternal/caregiver schooling was assessed based on the highest level of education attained. This variable was then transformed into years of schooling and stratified into three categories (≤ 7, 8−11, and ≥ 12). The number of residents *per* bedroom was assessed using the average number of individuals living in each household bedroom. Total monthly family income was divided by the number of household members to calculate *per capita* income, which was categorised into four groups based on fractions of the monthly minimum wage during the research periods (BRL 998.00 in 2019 and BRL 1045.00 in 2020): ≤ 0.25, > 0.25–≤ 0.50, > 0.50−≤ 1.00, and > 1.00. The USD exchange rate in December 2019 was used as a reference (BRL 4.013 = 1 USD).

The child's race/skin colour was determined based on the mother's/caregiver's declaration, with the categories being white, black, brown, yellow, and indigenous. The ‘yellow’ and ‘indigenous’ categories were excluded from the results due to their low prevalence and limited statistical power.

### 2.5 Statistical Analysis

The prevalence of FI levels was characterised according to sociodemographic variables, and each indicator's consumption and its 95% confidence intervals (95% CI) were estimated. Differences between the groups were considered statistically significant when no overlap in the 95% CI occurred. We also calculated the coefficient of variation (CV) of the estimates. CV is a measure of dispersion obtained from the ratio of the standard error to the estimated value for each indicator. We assumed that results with a CV of < 30% had adequate precision [29].

**Figure 1:**
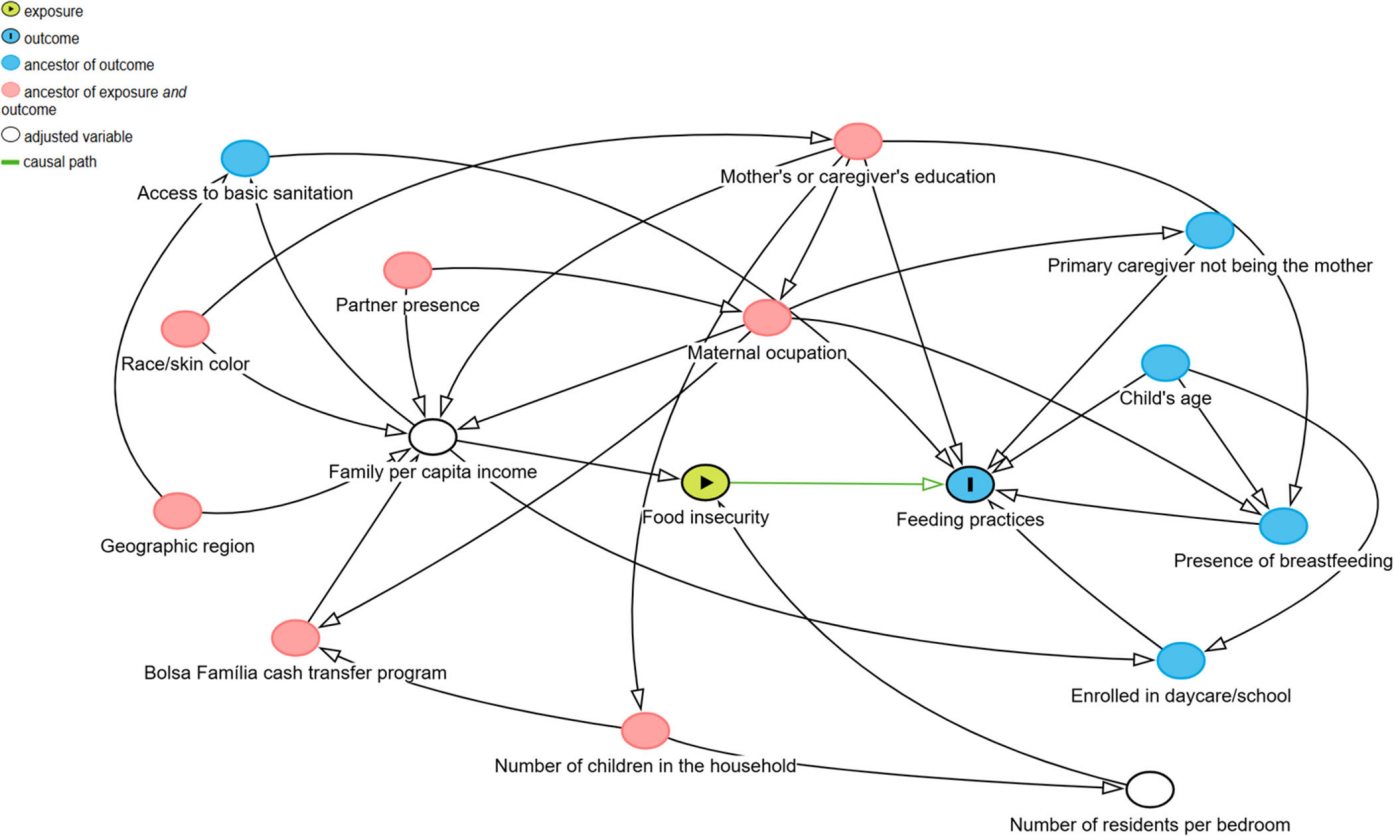
Directed Acyclic Graph representing the theoretical association between household food insecurity and feeding practices in Brazilian children under 59 months of age. Note: Minimal sufficient adjustment − number of residents per bedroom, per capita income.

A Directed Acyclic Graph (DAG) was constructed to represent the theoretical model and estimate the minimal sufficient adjustment of the association between FI and feeding practices in children < 5 y. DAG is a diagram used to determine dependency and independence between variables. All variables and associations included in the DAG must have sufficient scientific evidence that they do not occur by chance [30]. We included the DAG variables about the relation between FI and diet quality. The DAG was built using online DAGitty software [http://www.dagitty.net/dags.html] (version 3.0) [31]. The minimum set of variables suggested by the DAG included the number of residents per bedroom and family *per capita* income (Figure 1).

To estimate the association between feeding practices and FI, we performed a logistic regression for each indicator, adjusted by the minimally sufficient set of variables, and estimated the odds ratio (OR). We adjusted the *p*‐value from the regression analysis for multiple testing using the Benjamini–Hochberg method. A *p*‐value < 0.05 was considered statistically significant for all analyses.

Missing responses in the EBIA and in the structured feeding practices questionnaire were imputed using the hot deck method. For EBIA questionnaires that all questions were responded with ‘does not know/chose not to respond’, the donor observation was selected based on demographic and socioeconomic matching variables (household federal state, municipality, census enumeration areas income quartile, number of residents at the household, and sanitation conditions). In cases where only one or a few items were answered as ‘does not know/chose not to respond’, the donor was identified based on the available responses, with preference given to the donor whose answer matches the first item of the instrument. Data on the federal state and municipality were considered as less critical matching criteria.

For the structured feeding practices questionnaire, when responses were recorded as ‘does not know/chose not to respond’, donor observations were selected based on a set of matching variables: household federal state, municipality, census enumeration areas income quartile, child's age in months, mother's/caregiver's schooling, and breastfeeding status. The selected donor provided all missing values for the recipient record of this questionnaire; however, preserving the information provided by the family.

Statistical tests were performed in the R programming language (https://www.R-project.org/), using ‘srvyr’ and ‘survey’ packages to consider the structure of the sample design, weights, and calibration [32].

### 2.6 Ethical Aspects

Ethical approval was obtained from the Institutional Review Board of the Institutional Review Board of the Clementino Fraga Filho University Hospital of the Federal University of Rio de Janeiro (CAAE 89798718.7.0000.5257). Participation in the study began after the child's parent or caregiver signed two copies of the informed consent form.

## 3 Results

Most Brazilian children < 5 y lived in the Southeast (39.2%) and Northeast (28.1%) and had access to basic sanitation (73.0%). Most households had one or two residents (55.8%), and only 12.7% had per capita income > 1 minimum wage. Most mothers had 8–11 years of schooling (60.5%) (Table 1).

**Table 1:** Sample characteristics according to levels of food insecurity, Brazil, ENANI‐2019.

Variables	Sample characteristics		Prevalence of food insecurity					
			Mild		Moderate		Severe	
	% (95% CI)		% (95% CI)					
**Brazil**			37.8	33.3; 42.3	6.1	5.0; 7.1	4.2	3.3; 5.2
**Region**								
North	10.9	10.9; 10.9	43.4	38.8; 48.1	11.7	9.6; 13.8	7.0	3.0; 10.9
Northeast	28.1	28.1; 28.1	47.2	37.3; 57.1	6.6	4.6; 8.6	6.0	3.7; 8.3
Southeast	39.2	39.2; 39.2	33.0	24.4; 41.7	5.7	3.6; 7.8	2.6	1.5; 3.8
South	13.5	13.5; 13.5	31.8	26.4; 37.3	2.3	1.5; 3.1	3.2	1.2; 5.2
Midwest	8.3	8.3; 8.3	31.1	26.3; 35.9	4.8	3.3; 6.3	3.7	1.9; 5.4
**Access to basic sanitation**								
Yes	73.0	69.6; 76.3	36.1	31.1; 41.1	5.1	3.8; 6.5	3.5	2.6; 4.3
No	27.0	23.7; 30.4	42.6	37.4; 47.7	8.6	7.0; 10.3	6.3	4.1; 8.5
**Number of residents per bedroom**								
1−2	55.8	52.9; 58.7	35.2	29.9; 40.6	3.5	2.7; 4.3	2.7	1.9; 3.5
3	28.0	26.1; 29.8	42.6	37.0; 48.2	7.3	5.5; 9.2	3.7	2.2; 5.2
≥ 4	16.2	14.2; 18.3	38.4	33.8; 42.9	12.6	9.3; 15.9	10.2	6.7; 13.7
**Maternal schooling (years)**								
≤ 7	22.7	20.9; 24.6	31.9	4.9; 58.8	2.8	0.0; 7.8	16.6*	0.0; 40.6*
8−11	60.5	58.4; 62.5	37.4	32.5; 42.3	5.5	4.2; 6.8	3.2	2.3; 4.1
≥ 12	16.8	15.1; 18.5	30.5	24.7; 36.3	1.1*	0.4; 1.9*	1.7*	0.0; 3.5*
**Per capita income (fractions of minimum wage)**								
≤ 0.25	29.1	25.9; 32.2	43.3	38.2; 48.5	13.6	10.9; 16.2	10.3	8.2; 12.4
> 0.25−≤ 0.50	33.3	30.9; 35.7	43.0	36.9; 49.1	5.0	3.3; 6.8	2.8	1.8; 3.8
> 0.50−≤ 1.00	25.0	22.6; 27.4	31.0	24.9; 37.2	1.1	0.5; 1.7	1.0*	0.4; 1.6*
> 1.00	12.7	11.1; 14.2	25.1	19.9; 30.2	1.3*	0.0; 3.4*	0.1*	0.0; 0.3*
**Child′s age (months)**								
0−5	10.0	10.0; 10.0	35.6	30.1; 41.0	4.5	2.8; 6.2	4.6	2.9; 6.2
6−23	30.1	30.1; 30.1	36.5	31.8; 41.2	6.8	5.2; 8.3	3.1	2.3; 4.0
24−59	59.9	59.9; 59.9	38.9	34.1; 43.6	6.0	4.9; 7.1	4.7	3.5; 5.9
**Child race/skin colour**								
White	40.7	38.0; 43.3	33.2	28.8; 37.5	4.6	3.4; 5.8	3.0	2.1; 3.9
Brown	52.1	49.5; 54.7	40.8	35.5; 46.1	6.7	5.3; 8.2	4.8	3.4; 6.3
Black	6.6	5.5; 7.6	43.1	34.6; 51.7	8.8	4.4; 13.2	7.1	4.2; 10.0

*Note:* Statistical difference considering the lack of overlap of the 95%CI. Minimum wage: R$ 998.00 (~US$ 252.88) in 2019 and R$ 1039.00 (~US$ 247.90) in 2020. Basic sanitation: ‘yes’ whether the household had a water supply, sewage, and solid waste management.

* Coefficient of variation > 30%

The prevalence of FI was 48.1%, with 37.8% for mild, 6.1% for moderate, and 4.2% for severe form. The prevalence of moderate FI was highest in the North (11.7%) and lowest in the South (2.3%). Moderate and severe FI were more prevalent when the number of residents per bedroom was ≥ 4. In comparison, for households with 1–2 residents per bedroom, the prevalence of severe FI was 2.7%, and for families with 3 residents per bedroom, it was 3.7%. Higher overall prevalences of FI were observed in the lower per capita income categories, with a clear gradient: as income decreased, the prevalence of mild, moderate, and severe FI increased (Table 1).

**Figure 2:**
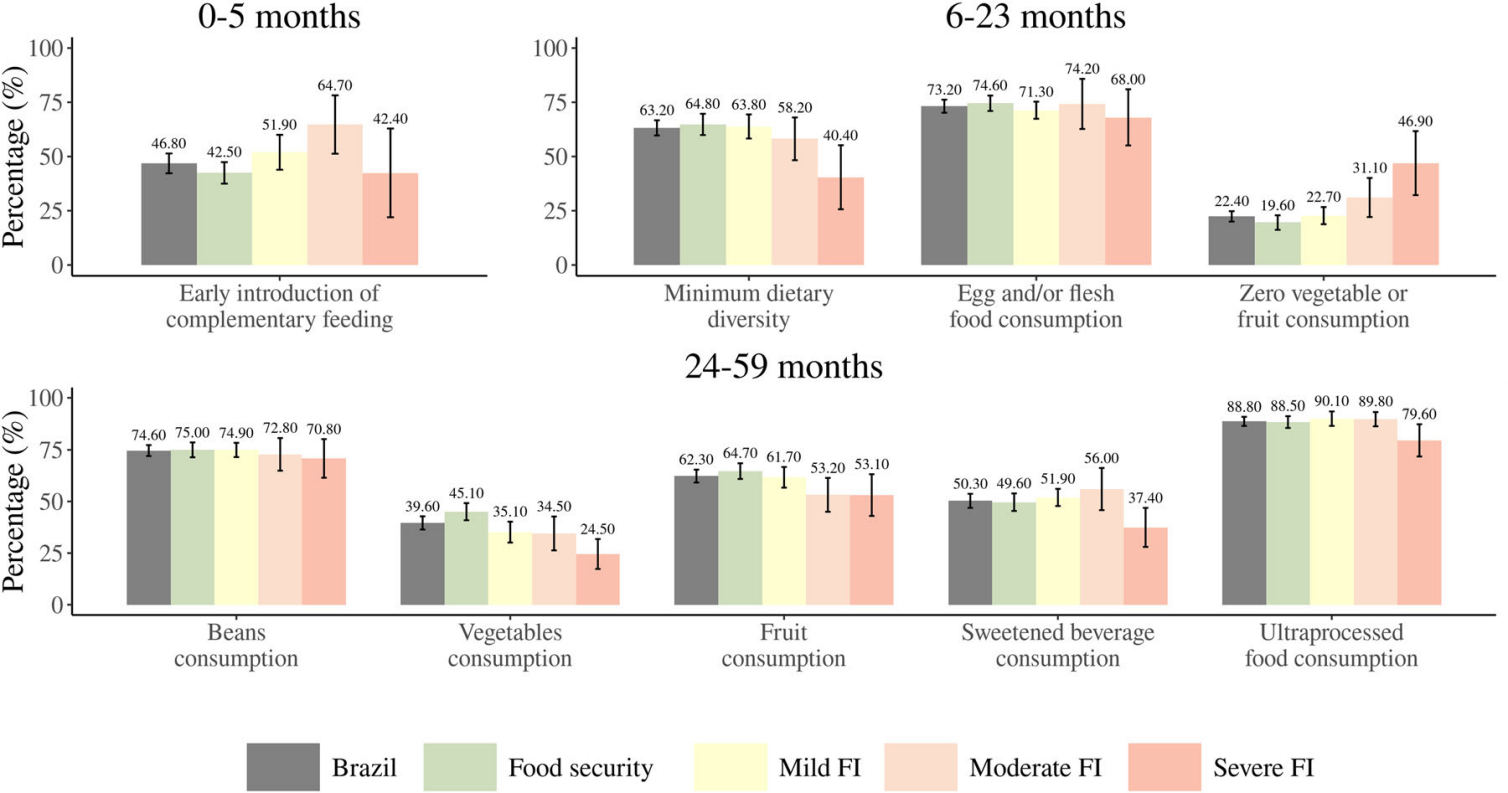
Prevalence of feeding practices markers across different age groups in Brazilian children under 59 months, Brazil, ENANI‐2019. Notes: Statistical difference considering the lack of overlap of the 95%CI. I 95% confidence interval. FI – food insecurity. a: statistically significant difference in the prevalence of food security compared to moderate FI. b: statistically significant difference in the prevalence of severe FI compared to food security and mild FI. c: statistically significant difference in prevalence of food security compared to mild FI and moderate FI. d: statistically significant difference in prevalence of severe FI compared to mild FI. Minimal dietary diversity − consumption of at least five of these eight food groups: (1) breast milk; (2) cereals, roots, and tubers; (3) legumes and seeds; (4) dairy products; (5) meats and liver; (6) eggs; (7) fruits and vegetables as a source of vitamin A; (8) other fruits and vegetables. Ultra‐processed foods − sweet or savoury biscuits/cookies, baby cereal, yogurt, sweets and treats, sausages and processed meats, packet snacks, packaged bread, and instant noodles. Sweetened beverages − soda, packed juice, packed coconut water, guarana syrups, redcurrant syrup, powdered juice, or fruit juice with added sugar.

Children < 6 months in the moderate FI households had a higher prevalence of early introduction of complementary feeding (64.7% [95% CI: 51.3−78.2]) when compared to the ones in food‐secure households (42.5% [95% CI: 37.5−47.4]). For children between 6−23 months in severe FI households had a lower prevalence of MDD (40.4% [95% CI: 25.7−55.2]) when compared to the ones in the mild FI and food‐secure households (63.8% [95% CI: 58.3−69.4] and 64.8% [95% CI: 59.9−69.7], respectively). Children aged 6‐23 months in the severe FI households had a higher prevalence of zero consumption of vegetables or fruits (46.9% [95% CI: 32.2−61.7]) when compared to the ones in mild FI and food‐secure households (22.7% [95% CI: 18.8−26.7] and 19.6% [95% CI: 16.2−22.9], respectively). For children between 24 and 59 months, children living in food‐secure households had a higher prevalence of vegetable consumption (45.1% [95% CI: 40.9−49.2]) when compared to the ones in the mild and severe FI households (35.1% [95% CI: 30.1−40.2] and 24.5% [95% CI: 17.3−31.8], respectively) (Figure 2).

In the adjusted regression model, we observed no significant association between FI and the early introduction of complementary feeding. However, children between 6 and 23 months experiencing severe FI had a 60% lower chance of achieving MDD (OR = 0.40; *p* = 0.026) and 2.48 times higher chance of consuming zero vegetables and fruit (OR = 2.48; *p* = 0.026), when compared to those experiencing food security. For children aged 24–59 months living with severe FI, the chance of consuming vegetables was 53% lower than that of children in food‐secure households (OR = 0.47; *p* = 0.012) (Table 2).

**Table 2:** Association between food insecurity levels and feeding practices markers across different age groups in Brazilian children under 59 months, Brazil, ENANI‐2019.

	0–5 months			6–23 months								
	Adjusted model^a^			Adjusted model^a^								
Food insecurity levels	Early introduction of complementary feeding			Minimum dietary diversity			Egg and/or flesh food consumption			Zero vegetable and fruit consumption		
	OR	95% CI	*p*‐value	OR	95% CI	*p*‐value	OR	95% CI	*p*‐value	OR	95% CI	*p*‐value
Food security	—			—			—			—		
Mild FI	1.31	0.88; 1.94	0.185	1.01	0.75; 1.38	0.934	0.94	0.72; 1.23	0.934	1.02	0.74; 1.41	0.934
Moderate FI	1.84	0.95; 3.55	0.070	0.82	0.52; 1.31	0.828	1.24	0.70; 2.21	0.828	1.32	0.78; 2.24	0.828
Severe FI	0.72	0.29; 1.78	0.472	0.40	0.21; 0.77	0.026*	0.97	0.52; 1.80	0.934	2.48	1.31; 4.69	0.026*

Food insecurity levels	24–59 months														
	Adjusted model^a^														
	Beans and/or other legumes consumption			Vegetables consumption			Fruit consumption			Sweetened beverages			Ultra‐processed food consumption		
	OR	95% CI	*p*‐value	OR	95% CI	*p*‐value	OR	95% CI	*p*‐value	OR	95% CI	*p*‐value	OR	95% CI	*p*‐value
Food security	—			—			—			—			—		
Mild FI	0.98	0.75; 1.29	0.912	0.72	0.54; 0.95	0.071	0.95	0.72; 1.24	0.744	1.10	0.89; 1.35	0.578	1.20	0.74; 1.96	0.622
Moderate FI	0.86	0.55; 1.34	0.633	0.76	0.49; 1.16	0.421	0.73	0.49; 1.10	0.371	1.28	0.83; 1.98	0.497	1.17	0.69; 2.00	0.639
Severe FI	0.79	0.47; 1.32	0.578	0.47	0.30; 0.73	0.012*	0.73	0.48; 1.11	0.371	0.60	0.39; 0.92	0.071	0.52	0.31; 0.88	0.071

*Note:* The *p*‐value was adjusted to multiple models using the Benjamini–Hochberg method.

a Adjusted to the number of children in the household and per capita income.

Minimal dietary diversity − consumption of at least five of these eight food groups: (1) breast milk; (2) cereals, roots, and tubers; (3) legumes and seeds; (4) dairy products; (5) meats and liver; (6) eggs; (7) fruits and vegetables as a source of vitamin A; (8) other fruits and vegetables.

Ultra‐processed foods − sweet or savoury biscuits/cookies, baby cereal, yogurt, sweets and treats, sausages and processed meats, packet snacks, packaged bread, and instant noodles.

Sweetened beverages − soda, packed juice, packed coconut water, guarana syrups, redcurrant syrup, powdered juice, or fruit juice with added sugar.

* *p*‐value < 0.05

## 4 Discussion

The ENANI‐2019 revealed a prevalence of 48.1% of food‐insecure households and 37.8% for mild, 6.1% for moderate, and 4.2% for severe FI among Brazilian children < 5 y. We observed that the prevalence of FI was higher in households with lower per capita income, larger family sizes, and without access to basic sanitation. Additionally, moderate and severe FI were more prevalent in the North region and among black children, with severe FI being more common in households with more residents per bedroom. Our findings also indicate no association between FI in different levels and early introduction of complementary feeding for children < 6 months. However, children aged 6–23 months experiencing severe FI had a 60% lower chance of meeting the MDD criteria and a 2.48 times higher chance of consuming no vegetables or fruit than those in food‐secure households. Additionally, among children aged 24–59 months, those living with severe FI had a 53% lower chance of vegetable consumption than their food‐secure counterparts.

The socioeconomic disparities observed in our study (region, residents for bedroom, per capita income, access to basic sanitation, and race/skin colour) have been widely documented in the literature, as evidenced by a systematic review published in 2020 that analysed 18 studies (2003–2018) on FI measured with the EBIA. The review found that FI was directly associated with socioeconomic and demographic indicators such as household head's education, geographic region, access to basic sanitation, and per capita income. Household size, as in the number of people living in the household, is also associated with FI. However, this relationship was mediated by per capita income, highlighting its crucial role in food access and dietary adequacy [7]. Another systematic review investigating social vulnerability and child FI in developed countries found a similar direction of association between overcrowded households and the prevalence of FI [33]. These findings highlight the urgent need for policies that address structural socioeconomic disparities to reduce FI. Future studies should investigate how interventions targeting overcrowded households and low‐income communities can improve access to adequate nutrition, especially for young children.

We observed no association between FI and the early introduction of complementary feeding. These results seem to be similar to the evidence found in the literature. Owais et al. [34] also found no difference in the prevalence of early introduction of complementary feeding between levels of household food security in children who completed a 9‐month follow‐up in rural Bangladesh. Ezzeddin et al. [35] analysed the association between exclusive breastfeeding (no early introduction of complementary feeding) and FI in the first 6 months of age of children who attended community health centres in West Tehran and found no association. These findings suggest that FI may not be a key determinant of the early introduction of complementary feeding. Other factors, such as place of residence, maternal age and education, maternal employment, child's age, and use of a pacifier, play a more significant role in exclusive breastfeeding duration and consequentially in the introduction of breastfeeding initialisation in Brazil [36].

Our results highlight that consuming fruits and vegetables for children aged 6−23 months and vegetables for those aged 24−59 months are critical for infant feeding practices in severe food‐insecure households. These results were expected and agree with other evidence in the literature. Duh‐Leong et al. [37] studied maternal and toddler participants in a New York public hospital (up to 19 months of age). They found that toddlers whose mothers experienced prenatal FI had a 6.3 times lower fruit and vegetable intake. Murillo‐Castillo [38] observed that children aged 6−12 years living in food‐insecure households in Mexico had significantly lower consumption of vegetables and fruits. Access to fruits and vegetables can be a challenge, especially for those experiencing FI. Low‐income households acknowledge the nutritional benefits of fruits and vegetables; however, they frequently perceive these foods as economically inaccessible, especially during periods of constrained financial resources, such as the end of the month. As FI intensifies, the perceived prohibitive cost of nutritious foods reduces parental purchasing of these items despite their intention to provide healthy meals for their children [39].

A 2015 study evaluating the economic impact of aligning dietary intake with the Brazilian dietary guidelines found that adhering to the recommendations would lead to an increase in food expenses of 58% among individuals with lower per capita incomes (≤ BRL 2.38/day) and 39% among those with higher per capita incomes (≥ BRL 13.83/day [BRL 415.00 – monthly minimum wage at the time of the study]). This was largely due to actual consumption exceeding recommendations for beans, oils/fats, sweets, and meat/eggs while falling short for fruits, vegetables, dairy, and grains [40]. Beyond the purchase cost, storing these highly perishable foods is also a concern. Compared to ultra‐processed foods, they have a significantly shorter shelf life, making their preservation and long‐term availability more challenging. As a result, dietary quality is compromised, increasing the risk of nutritional deficiencies and long‐term health consequences, such as anaemia and malnutrition [41, 42].

In contrast, a 2017–2018 study using national Brazilian data from the Consumer Expenditure Survey aimed to identify strategies for achieving dietary adequacy without raising food expenses. Verly‐Jr et al. [43] found that, although achieving full nutritional adequacy was challenging, certain feasible dietary adjustments, such as increasing the intake of fruits, vegetables, beans, tubers, dairy products, and nuts, could substantially improve diet quality and nutrient intake without additional costs.

In contrast, a 2017–2018 study using national Brazilian data from the Consumer Expenditure Survey aimed to identify strategies for achieving dietary adequacy without raising food expenses. The findings showed that it was difficult to achieve full nutritional adequacy; however, feasible changes could substantially improve dietary quality—increasing fruits, vegetables, beans, tubers, dairy products, and nuts—enhancing nutrient intake without additional costs [43].

FI negatively impacted the consumption of fruits, vegetables, and legumes but did not affect the intake of ultra‐processed foods in a 2013–2014 study conducted in Brazil [44]. This finding, which was observed a decade before our study, suggests that unhealthy dietary patterns were already prevalent among food‐insecure families, highlighting a possible long‐standing issue. Our results showed borderline but not statistically significant results for lower consumption of ultra‐processed foods and sweetened beverages among families with severe FI. Although not significant, these results diverge from other findings in the literature, such as Sharkey et al. [23], who reported that children aged 6–11 years residing in food‐insecure households had 377 additional calories and nearly 7 percent more calories from added sugar compared to food‐secure households. The mixed evidence on ultra‐processed food consumption in food‐insecure populations may reflect differences in age groups, cultural contexts, food systems, access to ultra‐processed foods, and coping strategies for FI adopted by families. Further research is needed to clarify these dynamics.

Given that FI restricts access to food, it can impact both the quantity and quality of the diets. As observed, this can reduce dietary variety, leading to the exclusion of specific food groups, such as fruits and vegetables, which affects the prevalence of MDD. Macharia et al. [22] found that children between 6 and 23 months in Kenya from families living in food‐secure households had 84% higher chance of achieving MDD than the ones living in FI. This result is consistent with our findings. Agbadi et al. [20] and Ahmed et al. [21] reported similar findings in Ghana and Ethiopia, studying children aged 6−23 months. They found a higher prevalence of MDD, that is, 45% and 57%, respectively—among children in food security, compared to those experiencing FI. In contrast, Bwalya et al. [45] found no association between MDD and FI for children aged 6–23 months in rural Zambia; it may be because they measured FI in 2 different dimensions—access and availability—and only the access influenced the MDD.

According to a 2021 FAO/WHO document, improving fruits and vegetables intake significantly reduces the prevalence of chronic diseases and thus lowers healthcare costs. The document highlights systemic changes, such as enhancing food accessibility and affordability, which are key to achieving long‐term public health benefits [46]. By implementing these strategies, healthcare systems can reduce costs related to preventable diseases, ultimately leading to more sustainable public health outcomes. Inadequate nutrition during childhood, often associated with FI, can lead to poor dietary habits that persist in adulthood, increasing the risk of noncommunicable diseases within families [47]. In Brazil, the economic impact of such conditions is substantial. Nilson et al. [48] showed that costs attributable to hypertension, diabetes, and obesity, including hospitalisations, outpatient care, and medication supplied by the public healthcare system, were estimated to exceed R$ 3.45 billion in 2018. This underscores the importance of promoting healthy diets early in life to support individual and societal health outcomes.

A Brazilian 2022 policy brief emphasises strategies such as promoting fruit and vegetable consumption through changes in food systems rather than solely viewing it as an individual choice. These strategies include subsidies to reduce costs, educational campaigns to encourage healthier eating, and strengthening food distribution programmes, like school meal programmes and food acquisition programmes, to increase access for vulnerable populations [49]. Additional studies are needed to explore various aspects of diet quality, such as dietary patterns and healthy diet index, among different FI categories, preferably using data from 24‐h dietary recalls. Moreover, future research should also aim to evaluate the effectiveness of these existing programmes in improving child‐feeding practices and reducing household FI, for example, by assessing their impact through nationally representative dietary data and programme coverage indicators. This approach would enable a more consistent investigation of dietary habits associated with FI, contributing to informed public policy decisions.

Using a structured questionnaire to assess food consumption based on a list of foods consumed the day before the interview may represent a limitation of this study. However, this method is widely used in literature to identify population‐level dietary differences effectively. Louzada et al. (2023) evaluated the questionnaire that served as the basis of our study and confirmed its effectiveness in capturing dietary intake nuances, reinforcing the validity of the method despite its limitations. In addition, the cross‐sectional design of the study limits the ability to establish causal relationships. There is also potential for recall bias, particularly in the collection of dietary data. While the sample was designed to be nationally and regionally representative, the findings may not fully capture the experiences of specific cultural or minority groups.

This study has strengths, such as the design with national representativeness and a validated scale to measure Brazilian household FI. The use of a national scale does not prevent comparison with other studies that use different existing scales, as noted by Marques et al. [50]. In a systematic review of different instruments used in epidemiological studies, it was observed that the common feature is that all instruments were developed from FI and hunger concepts linked to a lack of access to food due to scarce financial resources [50].

## 5 Conclusion

We observed differences in the prevalence of socioeconomic indicators across FI categories. Food‐insecure households had significant disparities in feeding, except among children < 6 months of age. Severe FI was consistently associated with poorer diet quality among children, particularly through reduced intake of fruits and vegetables and a lower likelihood of achieving minimum dietary diversity.

Addressing FI requires integrated public policies that ensure food quantity and promote access to diverse and nutritious foods. Interventions should prioritise low‐income families and those in overcrowded or underserved regions, focusing on early childhood nutrition. These may include low‐cost healthy food options, culturally appropriate meal planning strategies, and policies that enhance food access for vulnerable families. Future research should further investigate structural determinants of diet quality and evaluate targeted strategies such as subsidies, food environment improvements, and caregiver education that could mitigate the effects of FI on child nutrition.

## Author Contributions

**Juliana Mello:** conceptualisation (equal), formal analysis (lead), visualisation (lead), writing – original draft preparation (lead), writing – review and editing (equal). **Raquel Schincaglia:** data curation (equal), formal analysis (supporting), writing – review and editing (supporting). **Natália Oliveira:** formal analysis (supporting), writing – review and editing (equal). **Nadya Alves‐Santos:** project administration (equal), writing – review and editing (equal). **Paula Normando:** writing – review and editing (equal). **Dayana Farias:** formal analysis (supporting), writing – review and editing (equal). **Elisa Lacerda:** project administration (equal), writing – review and editing (equal). **Inês Castro:** project administration (equal), writing – review and editing (equal). **Gilberto Kac:** project administration (equal), resources (lead), supervision (lead), writing – review and editing (equal).

## Ethics Statement

This study was conducted according to the guidelines laid down in the Declaration of Helsinki and all procedures involving research study participants were approved by the Institutional Review Board of the Clementino Fraga Filho University Hospital of the Federal University of Rio de Janeiro (CAAE 89798718.7.0000.5257). Written informed consent was obtained from all subjects/patients.

## Conflicts of Interest

The authors declare no conflicts of interest.
